# Bioinspired Surfaces Derived from Acoustic Waves for On-Demand Droplet Manipulations

**DOI:** 10.34133/research.0263

**Published:** 2023-12-06

**Authors:** Zhuhao Wu, Lingyu Sun, Hanxu Chen, Yuanjin Zhao

**Affiliations:** ^1^ Department of Rheumatology and Immunology, Nanjing Drum Tower Hospital, School of Biological Science and Medical Engineering, Southeast University, Nanjing 210096, China.; ^2^ Oujiang Laboratory (Zhejiang Lab for Regenerative Medicine, Vision and Brain Health), Wenzhou Institute, University of Chinese Academy of Sciences, Wenzhou, Zhejiang 325001, China.; ^3^ Chemistry and Biomedicine Innovation Center, Nanjing University, Nanjing 210023, China.

## Abstract

The controllable manipulation and transfer of droplets are fundamental in a wide range of chemical reactions and even life processes. Herein, we present a novel, universal, and straightforward acoustic approach to fabricating biomimetic surfaces for on-demand droplet manipulations like many natural creatures. Based on the capillary waves induced by surface acoustic waves, various polymer films could be deformed into pre-designed structures, such as parallel grooves and grid-like patterns. These structured and functionalized surfaces exhibit impressive ability in droplet transportation and water collection, respectively. Besides these static surfaces, the tunability of acoustics could also endow polymer surfaces with dynamic controllability for droplet manipulations, including programming wettability, mitigating droplet evaporation, and accelerating chemical reactions. Our approach is capable of achieving universal surface manufacturing and droplet manipulation simultaneously, which simplifies the fabrication process and eliminates the need for additional chemical modifications. Thus, we believe that our acoustic-derived surfaces and technologies could provide a unique perspective for various applications, including microreactor integration, biochemical reaction control, tissue engineering, and so on.

## Introduction

The manipulation of droplets has attracted substantial attention across various fields, such as water transportation, water harvesting, and microreactor systems for chemical synthesis [1,2]. As a common strategy for droplet manipulation, functional surfaces have witnessed remarkable advancements owing to their customizable and versatile properties [3–9]. Given this, numerous functional surfaces with specific micro/nanostructures have been fabricated via advanced manufacturing techniques [10–14]. By means of chemical modification and multi-physical field (light and electricity) coupling, these surfaces can be endowed with capacity for effective transportation, control, and activation of droplets [15–22]. Actually, nature has evolved many surfaces with more powerful functions than imagined, which can serve as a guide for manufacturing and enriching the functionality of artificial surfaces for droplet control [23–28]. For example, structures on the flower and back of the desert beetle allow for effective water transportation and collection, respectively. However, it is difficult for the current artificial techniques to fully implement the droplet manipulation process of nature, owing to the complexity of surface fabrication and their limited functionality.

**Fig. 1.**
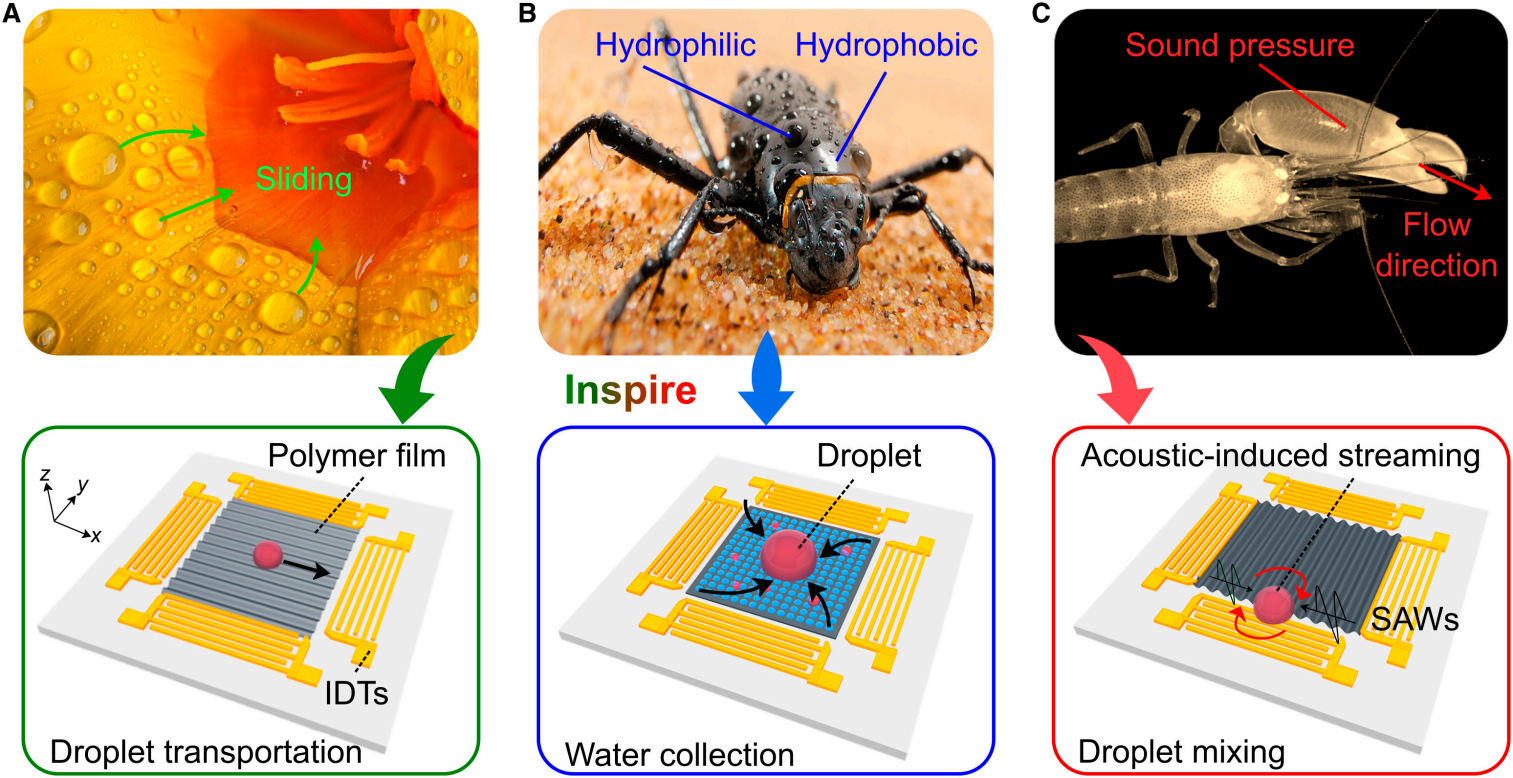
Bioinspired surfaces derived from acoustic waves for droplet manipulations. (A) Flower texture accelerates water droplet sliding, inspiring the acoustic fabrication of structures on polymer surfaces for regulating droplet transportation. Image courtesy of Simotion/iStock. (B) Acoustic fabrication of grid-like structures on polymer surface for water collection, inspired by the desert beetle. Image courtesy of Solvin Zanko/Minden Pictures. (C) Acoustic-induced streaming for accelerating the droplet mixing process, inspired by the pistol shrimp’s shooting. Copyright © 2021 Wei Wei, reprinted with permission from article (DOI: 10.1155/2021/9975952).

Here, we proposed a novel acoustic wave-based surface fabrication and manipulation approach to reproduce the droplet control manners in many natural creatures, as schemed in Fig. 1. Acoustic waves, as a form of mechanical vibration, can tailor the properties of material surfaces without the need for chemical treatments. Benefitting from this feature, acoustic-derived approaches has gained widespread popularity in both materials and life sciences [29–36]. For example, acoustic configurations can be used to facilitate the orderly patterning of polymer films, and engineer hydrogels with controllable bioadhesions [37–40]. Furthermore, acoustic-derived engineering approaches have become increasingly attractive in the multidimensional manipulation of droplets and fluids, due to their versatility, chemical-free nature, non-contact operation, activatability, and controllability [41–45]. These controlled droplets can be employed in the many biological applications, such as single-cell sequencing, digital PCR, point-of-care testing, and drug discovery [46–50]. Despite these advantages, few studies have reported the use of acoustic wave-based approaches for manufacturing wettability surfaces, and the practical values of acoustic-derived surfaces in complex droplet manipulations remain largely unexplored.

In this paper, we fabricated the wettability surfaces with the desired properties based on surface acoustic wave (SAW)-derived capillary waves. By adjusting the acoustic field, the generated capillary waves could deform various surfaces into pre-designed structures, such as parallel grooves and grid-like patterns. Because of the biomimetic features in structure and wettability, these microgrooved and grid-like surfaces have realized effective droplet transportation and water harvesting, respectively. Besides these static features, the derived surfaces with dynamic characteristics were also realized by using the acoustic wave-coupled technique. As surface roughness could be flexibly tuned by the SAW technique, the contact angle of water droplets could be programmably controlled on these dynamic surface textures. More attractively, the coupled acoustic waves on polymer surfaces were able to act as a stimulus to induce flow streaming inside droplets, resulting in accelerated chemical reactions. These results indicate that the proposed acoustic-derived platform offers a simple and new way to fabricate surfaces for controlling droplets, with significant potential in various biomedical applications.

## Results

**Fig. 2.**
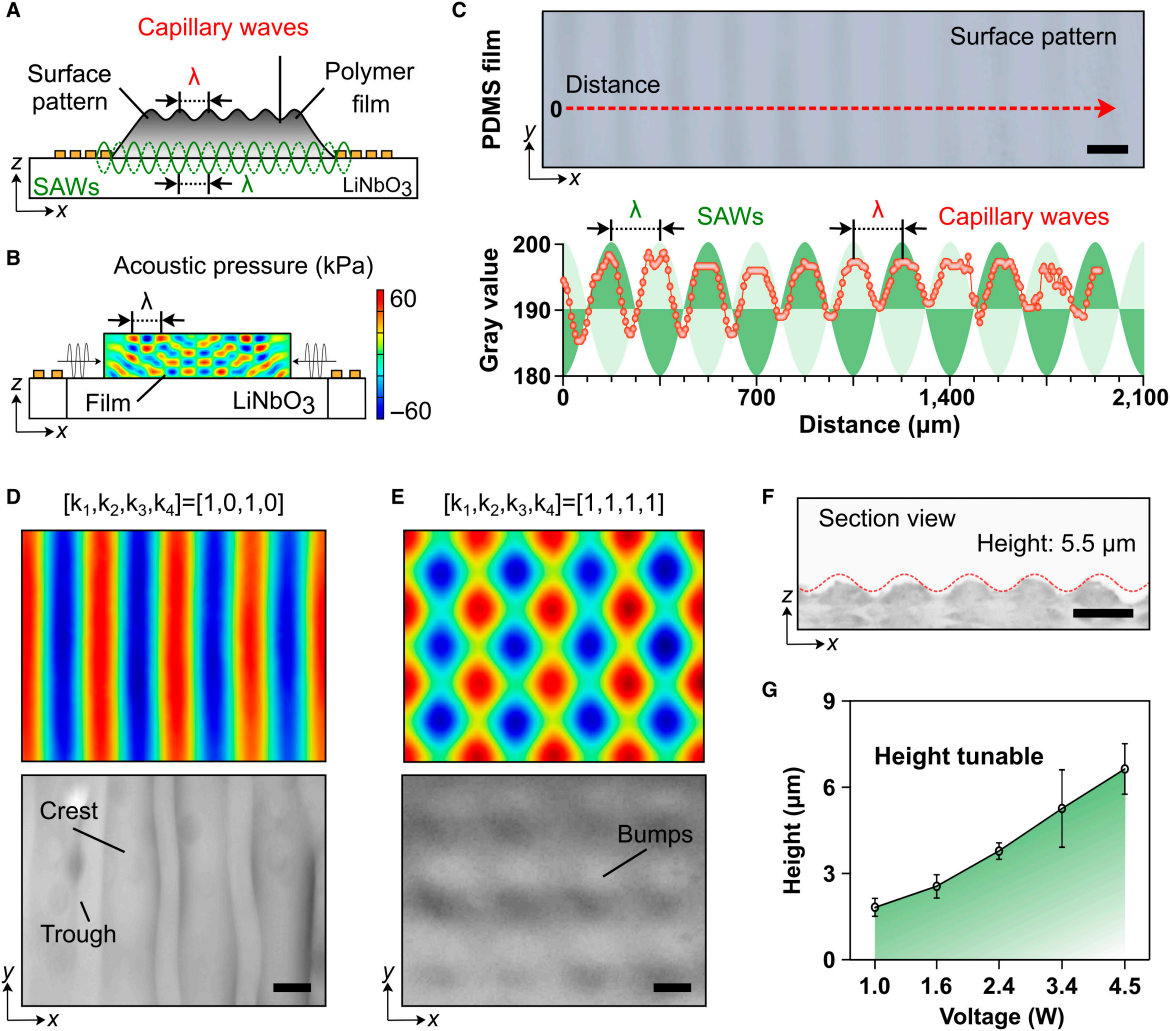
Acoustic manufacturing of material surfaces with periodic structures. (A) Schematic illustration of structures on polymer film fabricated by acoustic strategy. (B) Simulation of acoustic pressure distribution in polymer film. (C) Typical image showing the parallel structure on PDMS surface, matching the wavelength of SAW-induced capillary waves. (D) Parallel surface structure can be formed by activating a pair of parallel IDTs (k_1_ and k_3_). k_1_, k_2_, k_3_, and k_4_ represent the IDTs in counterclockwise order. (E) Grid-like surface structure can be formed by activating 2 pairs of IDTs (k_1_, k_3_ and k_2_, k_4_). (F) Section view of groove structure on PDMS surface. (G) Structure height can be tuned by the input signal power. Scale bar: 250 μm (D and E) and 10 μm (F), respectively.

In this study, we report on the development of a miniaturized acoustic device that enables the generation of acoustic-dependent microstructures on multifunctional surfaces (Fig. 2A). The experimental platform comprises a SAW generator and a 3-dimensional (3D)-printed chamber designed for loading materials with desired thickness (Figs. S1 and S2). By applying resonant radio frequency (RF) signals, SAWs are generated and propagated into polymer films, inducing capillary waves at the free surface of the polymer film. Capillary waves, characterized by uniform deformation and microstructure arrays, can be generated on various material films, ranging from fluids and hydrogels to elastomers, as shown in Fig. S3. As the capillary waves possess similar features, such as wavelengths, to those of the SAWs, the dimension of the fabricated microstructures can be controlled by the SAWs (Fig. 2B and C and Movie S1), and the detailed mechanism is shown in Figs. S4 and S5. We demonstrate the successful fabrication of parallel microstructures on thin polydimethylsiloxane (PDMS) film (Fig. 2D), and the use of changes in the acoustic field to fabricate microstructures with more complicated arrays on PDMS film (Fig. 2E and Fig. S6). The microstructure arrays were highly matched with the acoustic field distribution, and the section view revealed a sinusoidal structure that depended on the power of input signal (Fig. 2F). Moreover, we show that the height of the microstructure can be controlled by the power of RF signals (Fig. 2G). The deformation can be explained and predicted via the theoretical analysis (Supplementary Methods). Our findings demonstrate a novel and straightforward manufacturing strategy for the controllable production of microstructures on surfaces via acoustic waves.

**Fig. 3.**
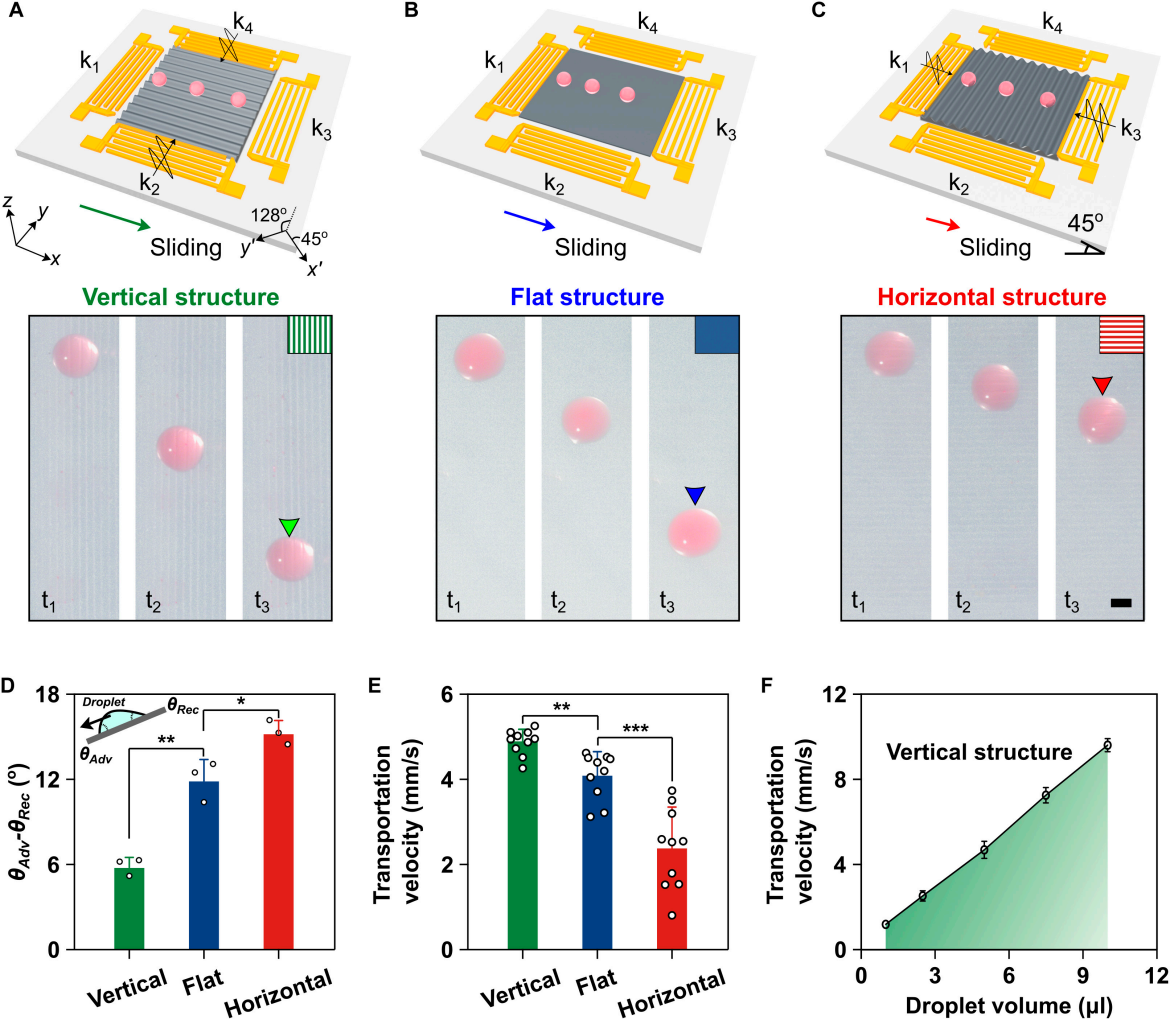
Acoustic manufacturing of microgroove structures on PDMS surfaces for controlling droplet transportation. (A) Schematics of vertical structure on PDMS surface can be formed by activating IDT k_2_ and k_4_, resulting in the acceleration of droplet sliding. The time interval from t_1_ to t_3_ is 0.25 s. We fixed the sloping angle of the device at 45° and the volume of water droplets at 2.5 μl in all next experiments. The right-bottom *x*′ and *y*′ axes showing the orientational cuts of the lithium niobate crystal and the IDTs’ position on the wafer. (B) Flat PDMS film with a height of 40 μm on SAW device for droplet sliding. (C) Schematics of horizontal structure on the PDMS surface can be formed by activating IDT k_1_ and k_3_, resulting in the deceleration of droplet sliding. (D) Analysis of the difference between the advancing angle (*θ_Adv_*) and receding angle (*θ_Rec_*) of a water droplet on PDMS surfaces with a vertical, flat, and horizontal structure. (E) Quantitative analysis of droplet transportation velocity on PDMS surfaces with a vertical, flat, and horizontal structure. (F) Dependence of transportation velocity on droplet volume. Scale bar: 150 μm.

To explore the effectiveness of acoustic-derived surfaces in regulating droplet transportation, we investigated the use of anisotropic PDMS surfaces with parallel microstructures to control the sliding velocity of water droplets. When a droplet is placed on a sloping surface, its velocity depends on the competition of wetting asymmetry and gravity on the droplet. By adjusting the direction of the structures against droplet transportation, the moving velocity of the droplet can be precisely controlled, as shown in Fig. 3A to C. We fixed the sloping angle of the device at 45° and measured the resistant force acting on a water droplet of 2.5 μl, which can be reflected from the difference between the advancing angle (*θ_Adv_*) and the receding angle (*θ_Rec_*). The force against droplet sliding was found to be smallest in the vertical structure (Fig. 3D), resulting in the fastest-moving velocity of droplet on this surface (Fig. 3E). In contrast, the surface with horizontal structure significantly slowed down the droplet sliding compared to the flat surface. Moreover, the droplet velocity was found to be linearly proportional to the droplet volume (Fig. 3F). The linear relationship can be demonstrated from the theoretical perspective (Supplementary Methods). These results indicate that acoustically manufactured microstructures on material surfaces can enable the on-demand manipulation of droplets.

**Fig. 4.**
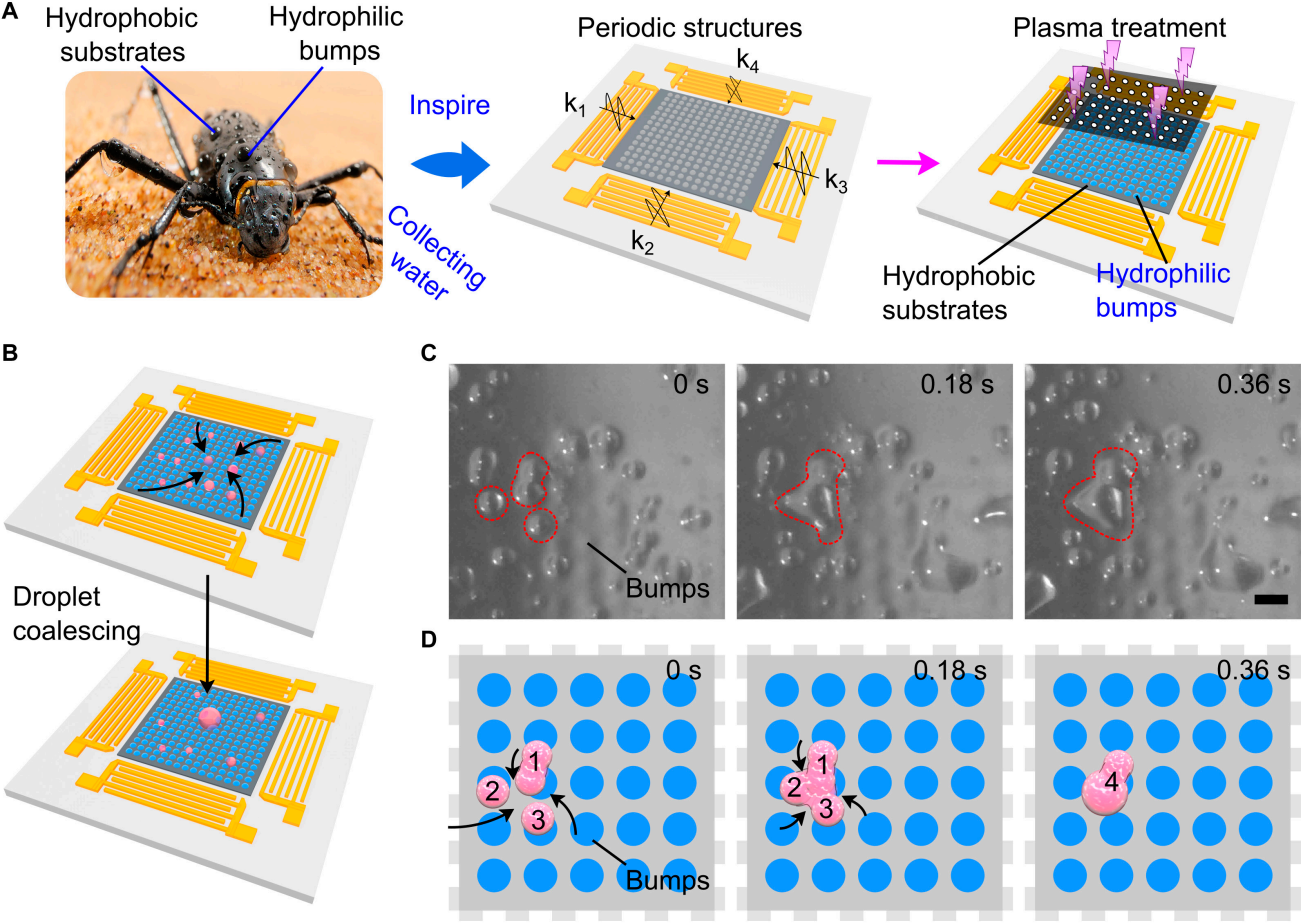
Acoustic manufacturing of grid-like structures on PDMS surfaces for water collection. (A) Schematics of the acoustic-assisted fabrication of desert beetle-inspired surfaces that have the periodic hydrophilic (blue region) and hydrophobic (grey region) distribution. The region exposed to plasma treatment showing the hydrophilicity. Image courtesy of Solvin Zanko/Minden Pictures. (B) Schematic illustration of droplet coalescence and growth on bioinspired functional surface. (C) In situ observation of the large water droplet formed from tiny droplets. (D) Schematic illustration of the large water droplet formed from tiny droplets. Scale bar: 500 μm.

With the integration of surface modification techniques, the acoustic wave-based surfaces could achieve more complicated wettability design for droplet manipulation. As a demonstration, we created hydrophilic/hydrophobic patterns on acoustic-fabricated surfaces, mimicking the structure and function of the desert beetle’s back (Fig. 4A). To obtain this specific surface, we firstly activated 2 pairs of interdigital transducers (IDTs) to generate a grid-like acoustic field, forming a periodic pattern on the PDMS surface. The cured PDMS film was treated with plasma under a 3D-printed mask (Fig. S7). After the plasma treatment, the exposed region of surface showed the hydrophilic property, while the unexposed region retained its original hydrophobicity, as shown in Fig. S8. Thus, the PDMS surface was structured and simultaneously possessed periodic hydrophilicity and hydrophobicity with the potential for water collection.

Then, we demonstrated the water droplet growth process on desert beetle-inspired surfaces to explore the underlying mechanism of water harvesting. Once water vapor was sprayed onto the processed surfaces, small droplets could spontaneously aggregate together and grow into big droplets, achieving the effective water-harvesting property (Fig. 4B). We recorded collection processes of water droplets in situ, as recorded in Fig. 4C. In a typical experiment, neighboring tiny droplets coalesced rapidly, leading to the formation of big droplet “1”, as shown in Fig. 4D. Immediately, the big droplet merged with tiny droplets “2” and “3”, achieving high-speed water harvesting (Movie S2). Together, these results indicate that the functional surface had excellent performance on water collection.

**Fig. 5.**
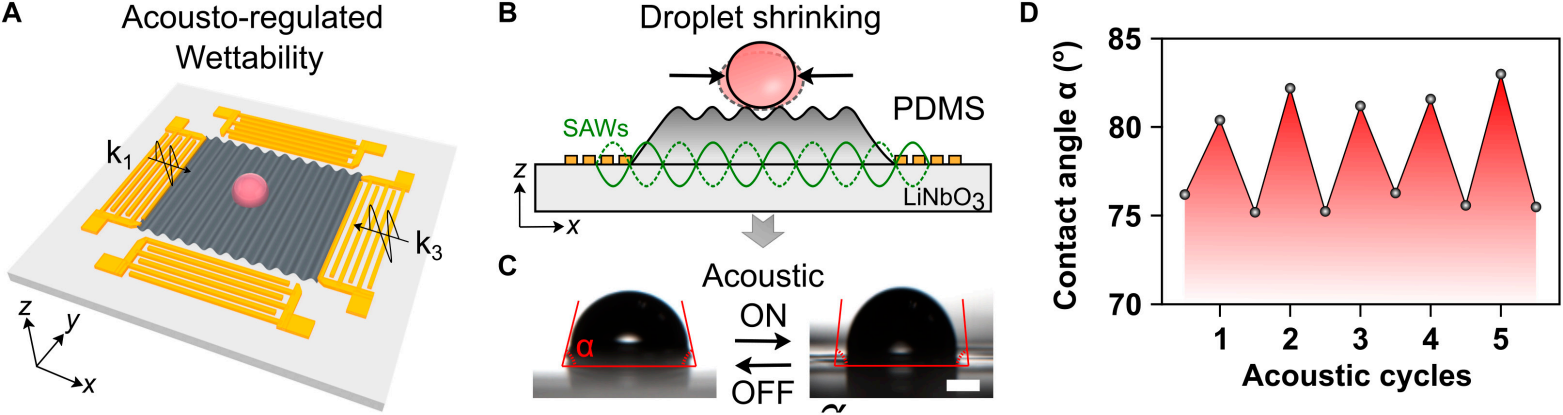
Programmable wettability enabled by acousto-regulated surface. (A) Schematic illustration of the surface roughness controlled by acoustic waves. (B) Schematics of droplet shrinking on acoustic-induced parallel textures on PDMS film. (C) Contact angle of water droplet on PDMS surface can be regulated by acoustic waves. The thin-layer PDMS coated on the piezoelectric substrate can be acoustically deformed into texture formats. (D) Droplet contact angle can be programmatically controlled with acoustic cycles. Scale bar: 500 μm.

Besides these static surfaces, our acoustic platform could also programmatically control the wettability of surfaces for dynamic manipulation of droplets, inspired by the pistol shrimp’s shooting, as shown in Fig. 5A. By activating the SAWs, parallel textures were generated on the PDMS surface, enabling precise tuning of the surface roughness (Fig. 5B). As a result, the droplet shrank into an equilibrium state with a smaller contact angle on the structured surface, as shown in Fig. 5C. Furthermore, the regulation process was found to be reversible by employing contactless acoustic waves. The contact angles of the water droplet were measured for 5 cycles with the acoustic waves on and off, demonstrating the cyclic reproducibility of the process, as shown in Fig. 5D and Movie S3. These results demonstrate that our acoustic platform enables tunable wettability on polymer surfaces, providing insights into on-chip droplet manipulations.

**Fig. 6.**
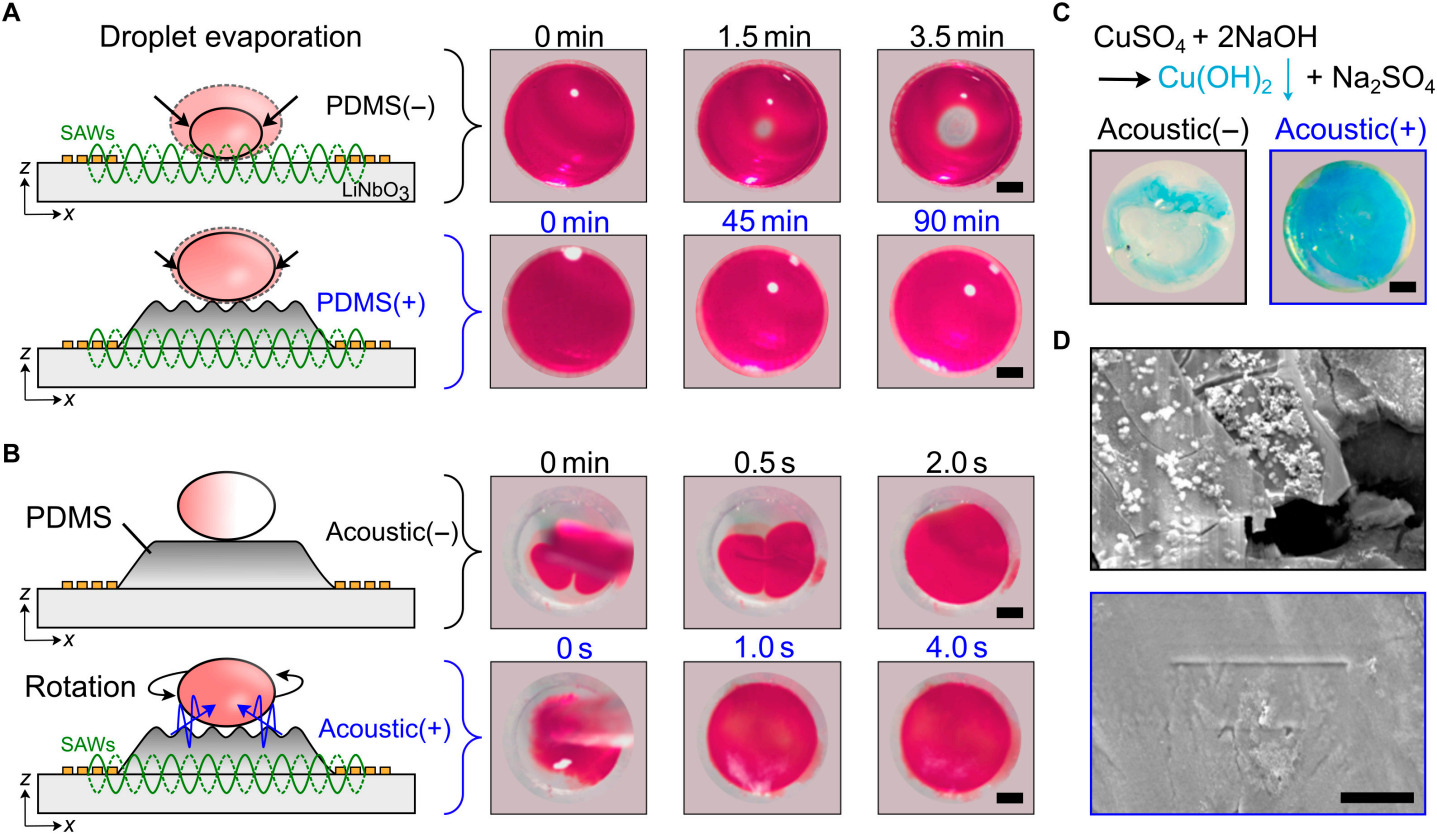
Acoustic-assisted multifunctional surfaces to manipulate droplets. (A) Evaporation suppression of droplets on PDMS-coated SAW device. (B) The acceleration of droplet mixing by acoustic activation demonstrated by simulation and experiments. (C) Acoustic-assisted micro-reactors, enabling the acceleration of chemical reaction and suppression of the coffee-ring effect. (D) SEM images showing the smoother surface of Cu(OH)_2_ precipitation via acoustic mixing. Scale bar: 50 μm.

To investigate practical applications of dynamic acoustic-derived functional surfaces, we integrated a droplet microreactor onto our acoustic platform with improved precision and efficacy of reactions. The PDMS coating on our acoustic platform significantly reduced droplet evaporation (Fig. 6A), ensuring the purity and concentration of reagents inside the microreactors. This phenomenon was due to the high energy absorption of PDMS film, where droplet volume remained stable for up to 1.5 h. In contrast, half of the liquid would evaporate after 3.5 min without the coating. In addition to increased accuracy, our acoustic functional surfaces also accelerated the mixing process inside the droplet microreactor. The schematics showed the vigorous streaming flow in droplets triggered by the acoustics, which was also observed in typical images of dye mixing where the mixing speed was at least 2 times faster with acoustic acceleration than without acoustic acceleration (Fig. 6B and Movies S4 and S5). As a model for demonstration, we used CuSO_4_ and NaOH aqueous solutions to synthesize Cu(OH)_2_ precipitation (Fig. 6C). Importantly, acoustic mixing overcame the coffee-ring effect in the droplet microreactor, resulting in a more uniform Cu(OH)_2_ product obtained under fast acoustic mixing (Fig. 6D and Movie S6). Our acoustic-assisted multifunctional surfaces have demonstrated the ability to stabilize and optimize the performance of droplet microreactors.

## Discussion

In summary, we have presented a novel and simple acoustic method for fabricating surfaces tailored for manipulating droplets with diameters ranging from 300 μm to 1.5 mm, inspired by the structures and functions of natural organisms such as flower, desert beetle, and pistol shrimp. A significant advantage of our approach is that it obviates the need of complicated fabrication process and additional chemical treatments to obtain functional surfaces. Besides, our technique provides a simplified and physical means of achieving on-demand droplet manipulations. Specifically, we have demonstrated the efficacy of SAWs in inducing capillary waves that can deform polymer films into pre-designed parallel and grid-like structures. These structured and functionalized surfaces exhibit impressive static abilities in droplet transportation and water collection. In addition, the robust regulation of surface wettability would be the synergistic effect of surface roughness and acoustic pressure distributions. Moreover, given the tunability and programmability of acoustics, the fabrication of polymer films can be dynamically controlled for optimal microreactor integration, including mitigating droplet evaporation, and accelerating chemical reactions. Benefitting from the wide-range dimension of droplet manipulations, our acoustic-driven surfaces can be further used in point-of-care testing like pathogen detection and diagnostics. These attributes suggest that the acoustic manufacturing of surfaces holds great promise for applications in diverse fields such as material bionics, energy science, and biomedical engineering.

## Methods

### Materials

PDMS and the polymerization component were purchased via Dow Corning Corporation. Dimethyl sulfoxide, poly(ethylene glycol) diacrylate (PEGDA) (Mw: 700), gelatin (from porcine skin), methacrylic anhydride (94%), rhodamine, sodium hydroxide, copper sulfate, and 2-hydroxy-2-methylpropiophenone were obtained through Aladdin. Methacrylate gelatin hydrogel was self-synthesized from gelatin and methacrylic anhydride. Ethanol was bought from Sinopharm Chemical Reagent Co., Ltd. Deionized water used for all the experiments was obtained from the Milli-Q Plus 185 water purification system (Millipore, Bedford, MA). The 128° YX-cut lithium niobate (LiNbO_3_) substrate was purchased from RDMICRO (Suzhou, China).

### Characterizations

Phase contrast images were acquired by using a microscope (Nikon ECLIPSE Ti2). Optical photos and frames were obtained from an Olympus stereomicroscope (BX51). The water contact angles were tested by using a machine (JC2000D2) at room temperature. High-resolution images were acquired from a scanning electron microscope (JSM-IT200, Japan).

### Fabrication of acoustic device

The SAW device was designed from the computer-aided design software and fabricated from the standard soft lithography. Figure S1 shows the optical image of the SAW device. Briefly, IDTs were manufactured by following the soft-lithography and metal membrane coating steps. IDTs in our acoustic platform were fabricated into 25 pairs of electrodes (*λ*/4 = 75 μm used in Figs. 2, 4, 5, and 6; *λ*/4 = 25 μm used in Fig. 3). These electrodes were deposited in 2 contacted layers (Cr layer: 10 nm; Au layer: 100 nm) on the LiNbO_3_ wafer using electron beam evaporation; the thickness of LiNbO_3_ substrate is 500 μm.

### Fabrication of film reservoir

The reservoir of polymer films was fabricated via the Projection Micro Stereo Lithography (PμSL) 3D printing system (nanoArch S140, BMF Material Technology Co., Ltd., China) with a resolution of 10 μm. The reservoir thickness was set as 40 μm for all experiments.

### Fabrication PDMS film with structures

The structuralized PDMS films were obtained from the rapid heat-induced curing under the acoustic deformation. Typically, PDMS (agent A:B = 1:8) was added into a reservoir above the SAW device. After applying an RF signal with a 150 mVpp voltage to the IDTs, a set of SAWs was produced, resulting in the generation of capillary waves. Once PDMS deformed by capillary waves was stable dynamically, the SAW device was placed onto a heater with a temperature of 90 °C. After 30 s, thin PDMS film was cured completely with the acoustic signal on and removed away from the heater and used in subsequent experiments. Notably, we selected the center region because there existed uniform structure patterns for demonstrating the formation of deformation patterns in all experiments.

### Water collection experiment

Tiny water droplets were used and sprayed for testing water collecting performance on the beetle-inspired PDMS film. Micrometer water drops were formed by a facial sprayer on functional films. The real-time record was performed under a microscope to observe the in situ coalescence of droplets.

### Statistical analysis

All the data were displayed as mean ± standard deviation. All results were statistically analyzed under the unpaired and 2-sided *t* tests using Prism 9 (GraphPad, La Jolla, CA). The statistical significance was set as: *P < 0.05, **P <0.01, and ***P < 0.001.

## Data Availability

The authors confirm that the data supporting the findings of this study are available within the article and/or its Supplementary Materials.
