# Supplementary material for: Bioinspired Surfaces Derived from Acoustic Waves for On-Demand Droplet Manipulations
Source: Research (Wash D C). 2023 Dec 6;6:0263. doi: 10.34133/research.0263 (PMC11407685; doi:10.34133/research.0263)
Supplement: Supplementary 1 — Supplementary Methods Figs. S1 to S8 [file research.0263.f1.zip › RESEARCH_D2301028f1.docx]

**Supplementary Methods:**

**Demonstration of SAW-induced capillary waves that deform polymer films.** Capillary waves are tiny surface-water waves. They have a very short wavelength, resulting in a rounded crest and a V-shaped trough because the water's surface tension acts as their restoring force. Capillary waves can be created on thin films when they are subjected to surface acoustic waves (SAWs), causing them to deform due to the balance of viscous and capillary forces [1, 2]. To predict the height of this deformation, here, we have developed a simplified physical model that takes into account the unique behavior of waves in thin films with high viscous stresses, low inertial forces, and surface tension effects. The governing equations for thin films are derived from the Navier-Stokes equations and can be simplified as follows:

$$\begin{aligned} \rho\left( \frac{\partial\boldsymbol{u}}{\partial t}+\boldsymbol{u}\cdot\nabla\boldsymbol{u} \right)=-\nabla p+\rho\boldsymbol{g}+\mu\nabla^{2}\boldsymbol{u}\#\left( 1 \right) \end{aligned}$$

The relevant parameters are: $\rho$, the density; $\boldsymbol{u}$, the velocity; $p$, the pressure; $\boldsymbol{g}$, the gravitational constant; and μ, the dynamic viscosity. The fluid is steady, so:

$$\begin{aligned} \frac{\partial\boldsymbol{u}}{\partial t}=0\#\left( 2 \right) \end{aligned}$$

The second term ($\boldsymbol{u}\cdot\nabla\boldsymbol{u}$) is can be disregarded due to the small ratio of ${V^{2}}/L$, where $V and L$ are the small characteristic velocity and length, respectively.Thus, without these inertial terms, the Navier-Stokes equation can be simplified as:

$$\begin{aligned} \nabla p=\rho\boldsymbol{g}+\mu\nabla^{2}\boldsymbol{u}\#\left( 3 \right) \end{aligned}$$

At the bottom of films, where the liquid meets the piezoelectric substrate, a no-slip condition is in effect. However, the velocity changes quickly in the *z* direction, resulting in a greater second derivative in that direction than in the *x* and *y* directions. This leads to a simplified equation as below:

$$\begin{aligned} \nabla p=\rho\boldsymbol{g}+\frac{\left( \partial\boldsymbol{u} \right)^{2}}{\partial z^{2}}\#\left( 4 \right) \end{aligned}$$

The velocities perpendicular to the plane are insignificant, thus:

$$\begin{aligned} \frac{dp}{dx}=\frac{\left( \partial u \right)^{2}}{\partial z^{2}}\#\left( 5 \right) \end{aligned}$$

$$\begin{aligned} \frac{dp}{dy}=\frac{\left( \partial u \right)^{2}}{\partial z^{2}}\#\left( 6 \right) \end{aligned}$$

$$\begin{aligned} \frac{dp}{dz}=\rho g\#\left( 7 \right) \end{aligned}$$

Here, we can employ the conversation of mass. The volumetric flow in the *x* direction, $\Delta Q=\left( \int_{0}^{h(x)} udz \right)_{x}^{x+dx}$, which must equal the decrease in the *z* direction ($-\frac{\partial h}{\partial t}dx$), as shown in Fig. S4. Equating both volumetric flows gives the following equation:

$$\begin{aligned} \frac{\partial Q}{\partial x}=\frac{\partial}{\partial x}\int_{0}^{h\left( x \right)} udz=-\frac{\partial h}{\partial t}\#\left( 8 \right) \end{aligned}$$

Integrating equation (5), (6), and (7) twice, with no slip at the wall and no shear at the liquid-gas interface, yields the following thin film horizontal velocity profile:

$$\begin{aligned} u=\frac{1}{\mu}\frac{\partial p}{\partial x}\left( \frac{z^{2}}{2}-hz \right)\#\left( 9 \right) \end{aligned}$$

Applying equation (8) to this velocity relationship gives the dynamic height of film as:

$$\begin{aligned} \frac{\partial h}{\partial t}=\frac{1}{3\mu}\frac{\partial}{\partial x}\left( h^{3}\frac{\partial p}{\partial x} \right)\#\left( 10 \right) \end{aligned}$$

**Theoretical simulation of acoustic pressure distributions.** The finite element package COMSOL 6.0a (the COMSOL Group) was used to implement the model, which followed our previous work [3]. As seen in **Figure S5**, two domains were the LiNbO_3_ substrate and water films. The thickness of LiNbO_3_ substrate was set as 500 μm, while the water films were set as 20, 40, 60, and 80 μm. The bottom boundary of water films was solid substrate, and the surrounding boundary was hard PMMA material. Two pairs of IDTs were placed around the water film domain. The numerical procedure began by selecting ‘Solid Mechanics’ and the ‘Electrostatics’ physics to calculate the vibration of piezoelectric substrate, which was governed by:

$$\begin{aligned} T_{ij}=C_{ijkl}^{E}\cdot S_{kl}-E_{k}\#\left( 11 \right) \end{aligned}$$

$$\begin{aligned} D_{i}=e_{ikl}\cdot S_{kl}+\varepsilon_{ij}^{S}\cdot E_{k}\#\left( 12 \right) \end{aligned}$$

where the stress vector is represented by $T_{ij}$, the strain vector is represented by $S_{kl}$, the electrical displacement is denoted as $D_{i}$, the piezoelectric matrix (C m^-2^) and permittivity matrix (F m^-1^) are denoted as $e_{ikl}$ and $\varepsilon_{ij}$, respectively. To facilitate this analysis, electrodes in IDTs were subjected to both positive and negative electric potential boundary conditions. Furthermore, perfectly matched layers and low-reflecting boundaries were implemented at the four sides of water films and at the bottom of the piezoelectric substrate to minimize reflections. The acoustic field in the water film domain was solved using the 'pressure acoustic' physics method. The problem of analyzing the harmonic, linearized acoustic field can thus be defined as:

$$\begin{aligned} \nabla^{2}p=-\frac{\omega^{2}}{c^{2}}p\#\left( 13 \right) \end{aligned}$$

where $p$ represents the complex pressure, which is defined at position $r$. And $p$ can be written as follows:

$$\begin{aligned} p_{t}\left( r,t \right)=Re\left\{ p\left( r \right)e^{-j\omega t} \right\}\#\left( 14 \right) \end{aligned}$$

To facilitate the analysis of the interaction between the physics of Solid Mechanics and Pressure Acoustics, several boundary conditions were applied. The top and four sides of the films were assigned as perfect match layers to PMMA boundary, while a velocity continuity boundary condition was imposed on the substrate surface. A Frequency Domain solver was then utilized to solve for all the aforementioned physics at a driving frequency of 13 MHz. As a result, variations in acoustic pressure distributions were observed, which were found to be dependent on the thickness of the films.

**Supplementary Figures:**


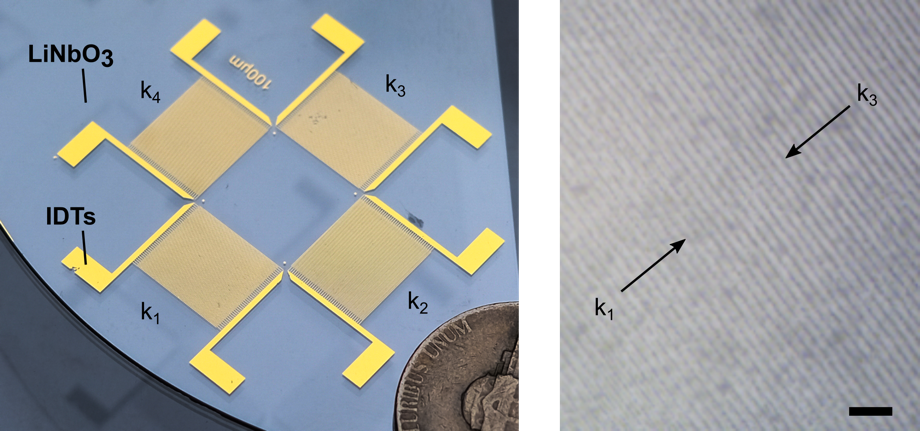


**Figure S1.** The typical image of surface acoustic wave device. k_1_, k_2_, k_3_, and k_4_ represent four interdigital transducers (IDTs). The typical pattern showing the result under the relative working resonant frequency (39.85 MHz) and input power (1 W). Scale bar: 200 μm.


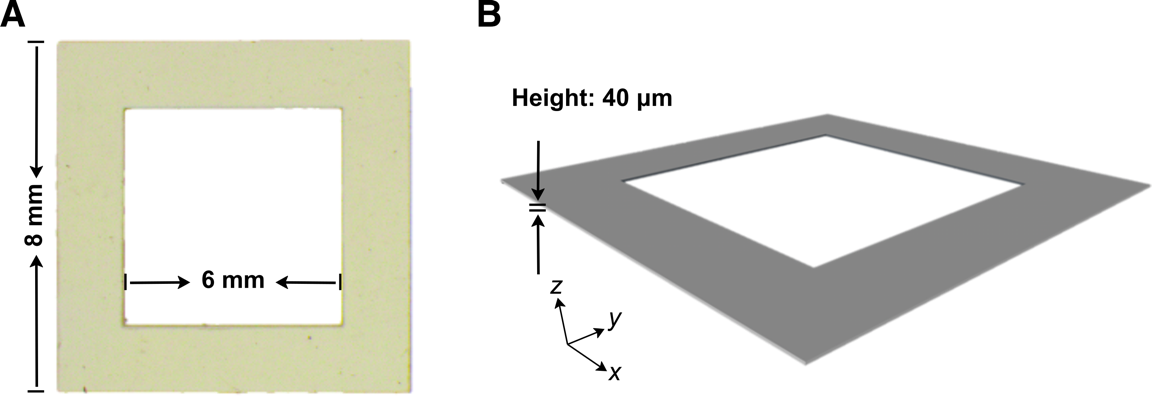


**Figure S2.** Design and device of the reservoir for loading polymer precursors. (**A**) The width and length of reservoir. (**B**) The height of reservoir


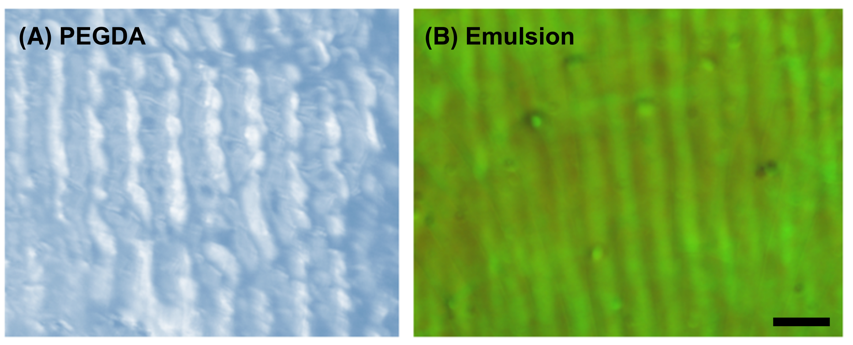


**Figure S3.** The general acoustic manufacturing of various material films. (**A**) The PEGDA film with parallel structures. (**B**) The emulsion with parallel patterns of colorized nanoparticles. Scale bar: 300 μm.


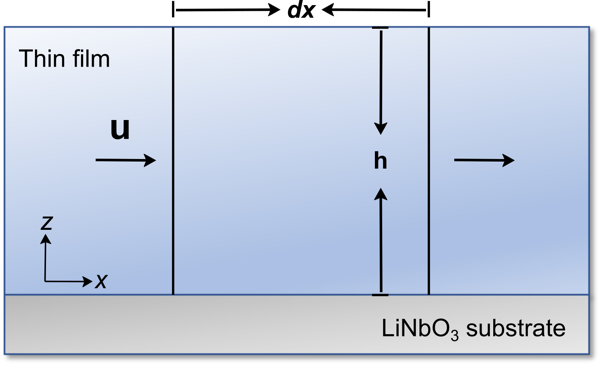


**Figure S4.** Demonstration of the role of capillary waves in film deformation: Schematics of flow in the thin film.


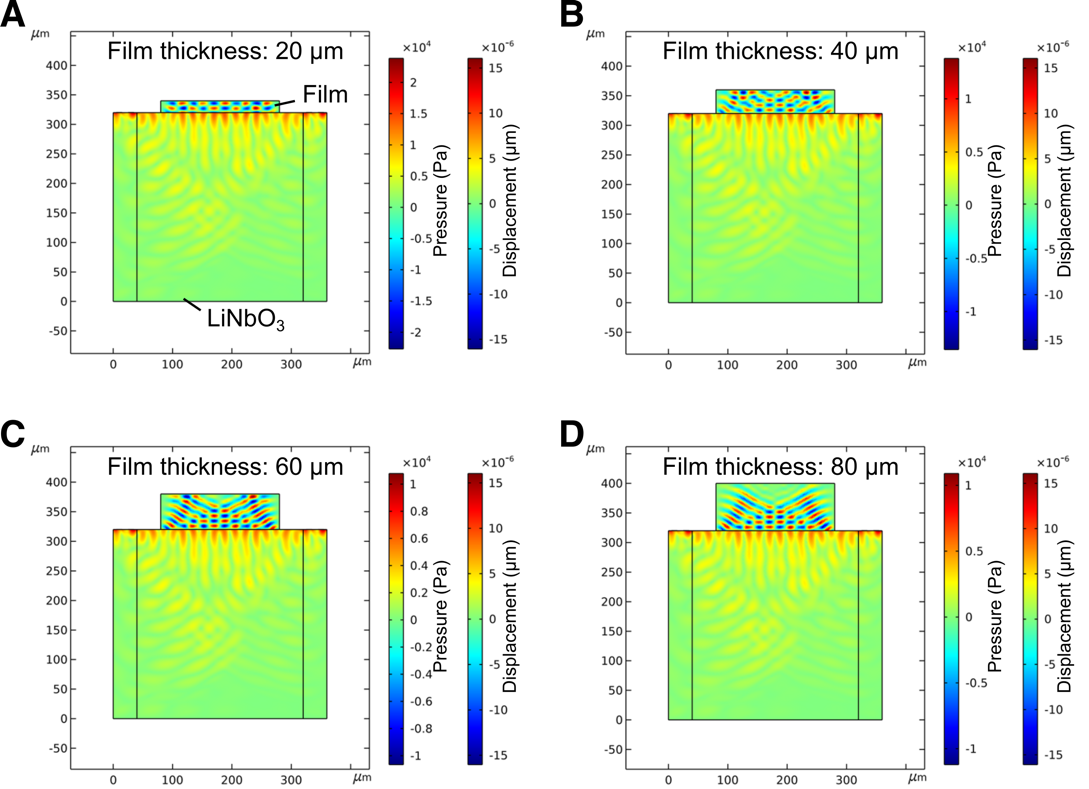


**Figure S5.** Simulation of acoustic pressure distributions in film with various thicknesses. (**A**) Thickness: 20 μm. (**B**) Thickness: 40 μm. (**C**) Thickness: 60 μm. (**D**) Thickness: 80 μm. All the voltages of input signal are 1 Vpp.


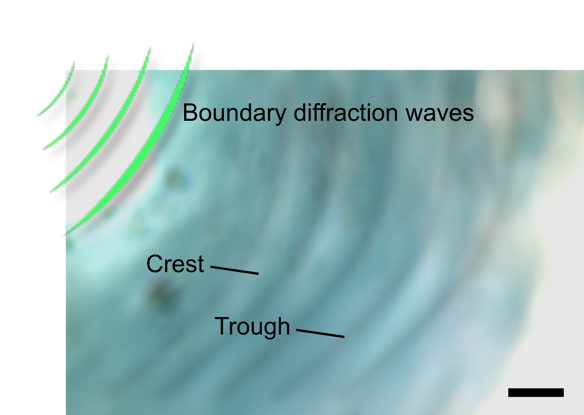


**Figure S6.** Diffraction-based acoustic fields for generating circle structure on film. Scale bar: 500 μm.


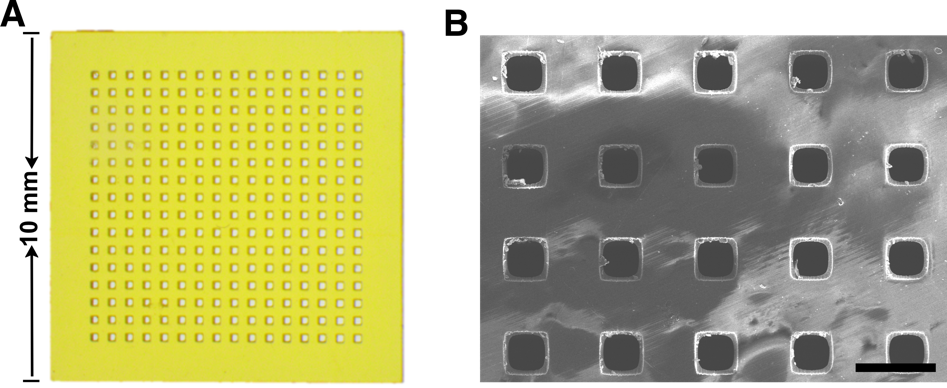


**Figure S7.** Design and device of the mask for changing the wettability of PMDS films. (**A**) The brightfield image of the mask. (**B**) The SEM image showing the design detail of the mask. Scale bar: 500 μm.


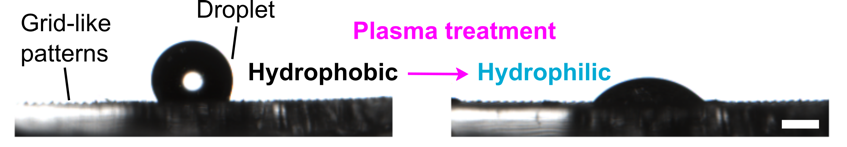


**Figure S8.** The wettability of PDMS film was changed from hydrophobic to hydrophilic via plasma treatment. Scale bar: 500 μm.

**Supplementary references**

1. Qi, A.; Yeo, L. Y.; Friend, J. R., *Physics of Fluids* **2008,** *20* (7), 074103. DOI 10.1063/1.2953537.

2. Tan, M. K.; Friend, J. R.; Matar, O. K.; Yeo, L. Y., *Physics of Fluids* **2010,** *22* (11), 112112. DOI 10.1063/1.3505044.

3. Wu, Z.; Cai, H.; Ao, Z.; Nunez, A.; Liu, H.; Bondesson, M.; Guo, S.; Guo, F., *Anal Chem* **2019,** *91* (11), 7097-7103. DOI 10.1021/acs.analchem.9b00069.
