# Supplementary figures and images for: Bioinspired Surfaces Derived from Acoustic Waves for On-Demand Droplet Manipulations
Source: Research (Wash D C). 2023 Dec 6;6:0263. doi: 10.34133/research.0263 (PMC11407685; doi:10.34133/research.0263)

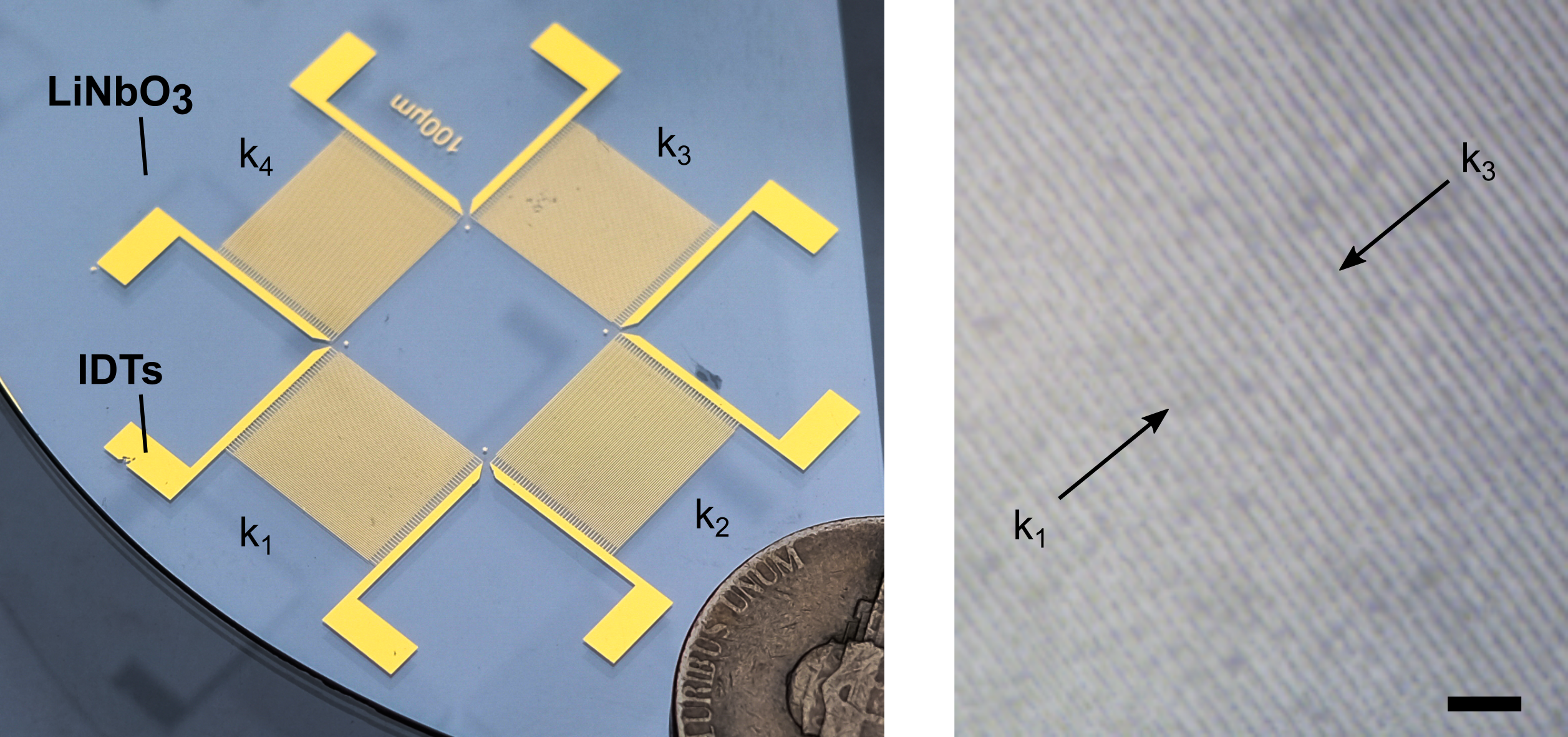

Supplement: Supplementary 1 — Supplementary Methods Figs. S1 to S8 [file research.0263.f1.zip › figs1.png]

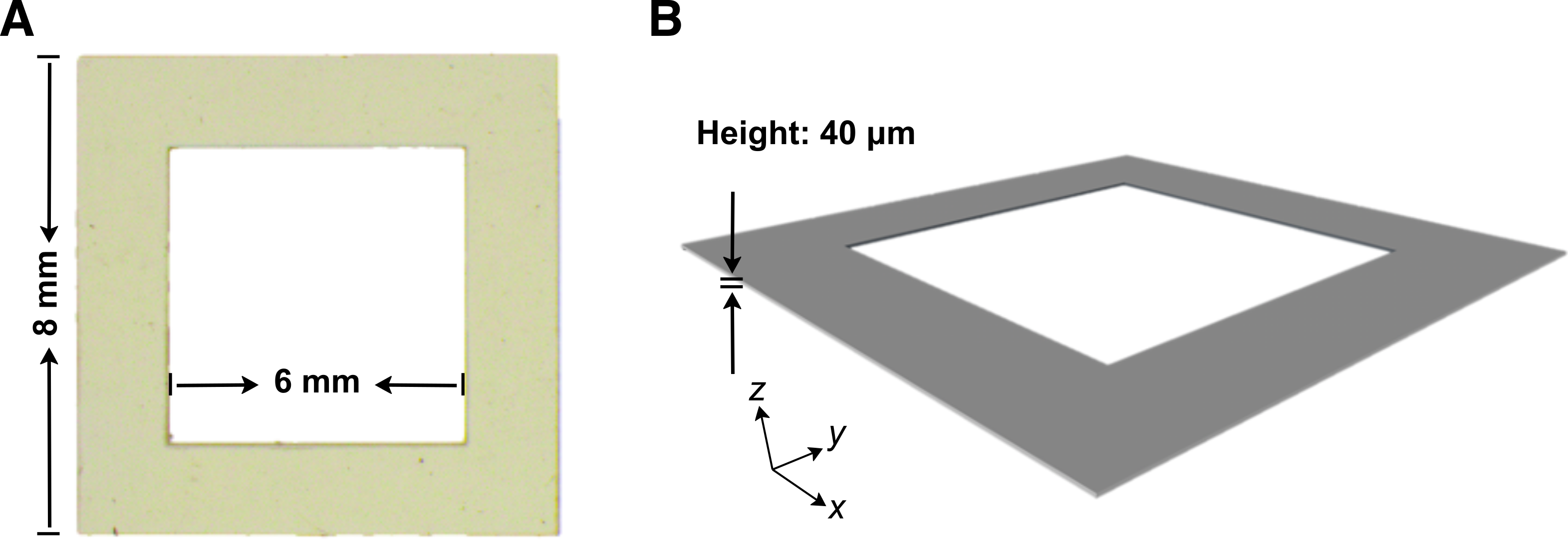

Supplement: Supplementary 1 — Supplementary Methods Figs. S1 to S8 [file research.0263.f1.zip › figs2.png]

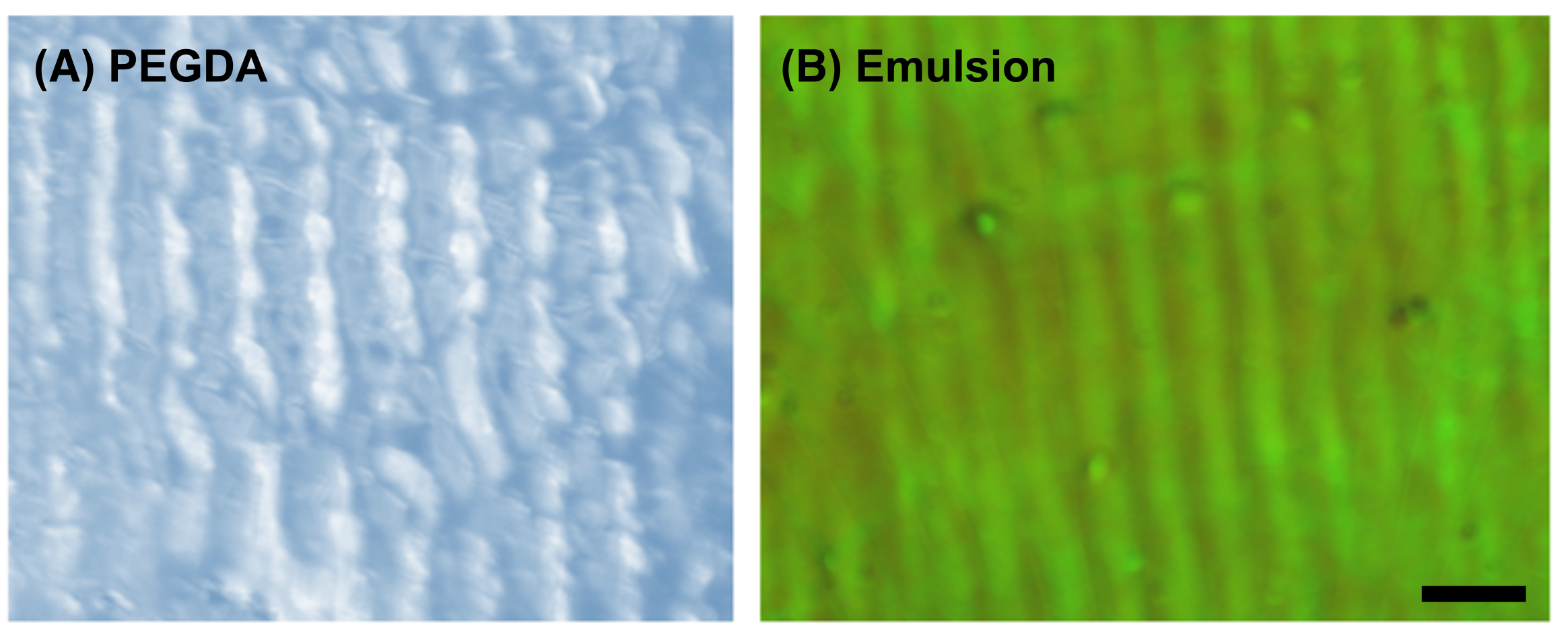

Supplement: Supplementary 1 — Supplementary Methods Figs. S1 to S8 [file research.0263.f1.zip › figs3.png]

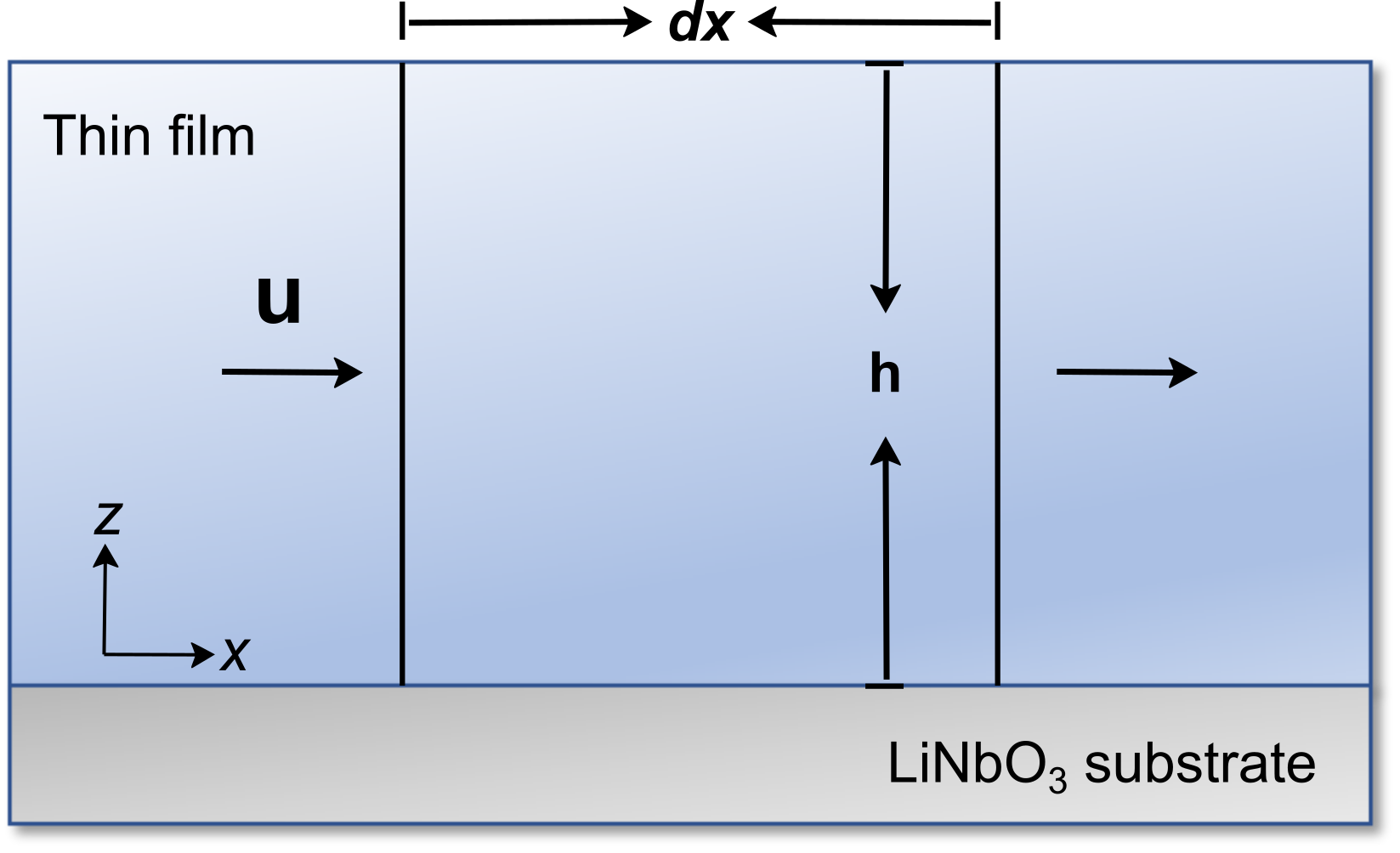

Supplement: Supplementary 1 — Supplementary Methods Figs. S1 to S8 [file research.0263.f1.zip › figs4.png]

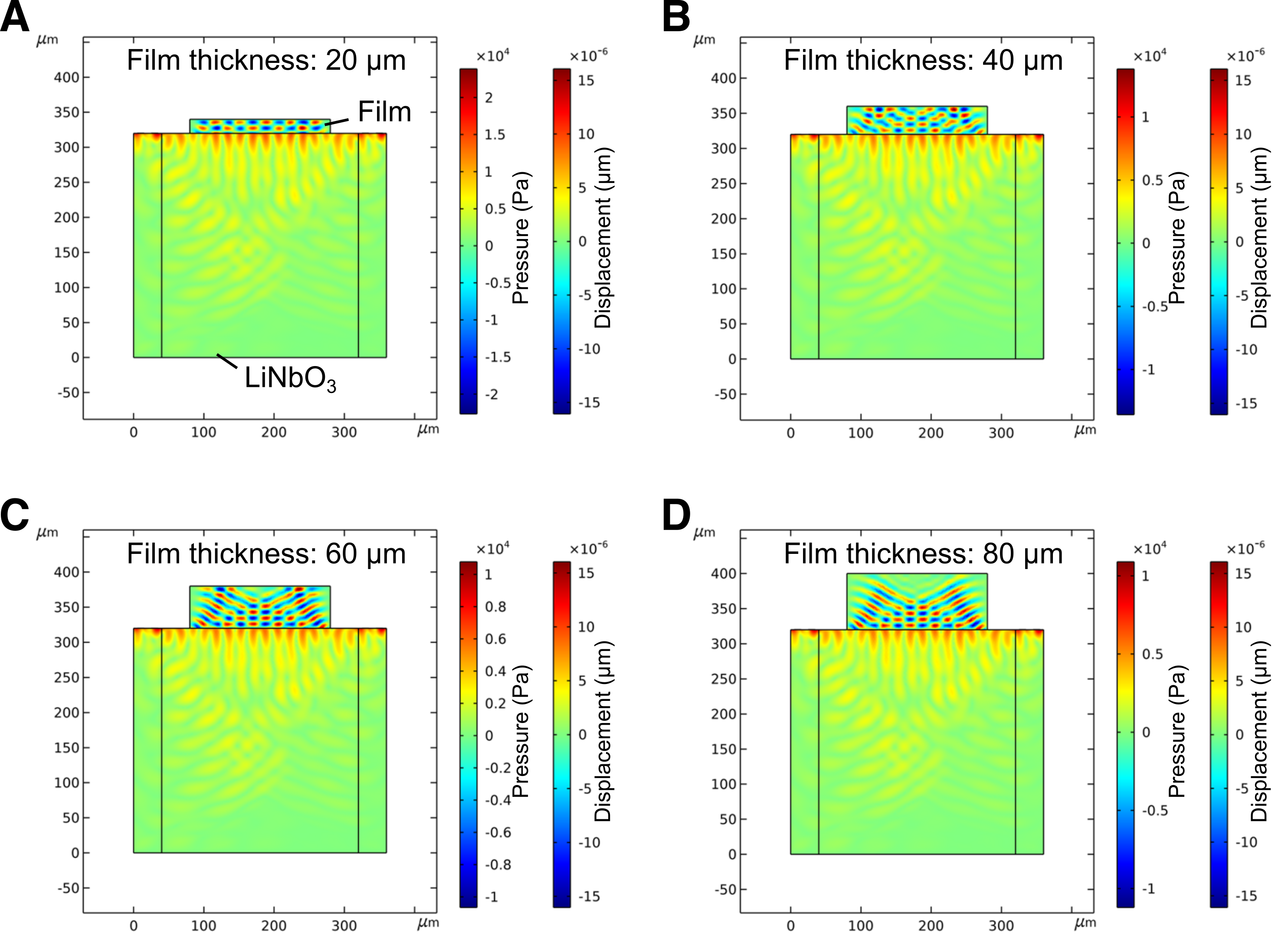

Supplement: Supplementary 1 — Supplementary Methods Figs. S1 to S8 [file research.0263.f1.zip › figs5.png]

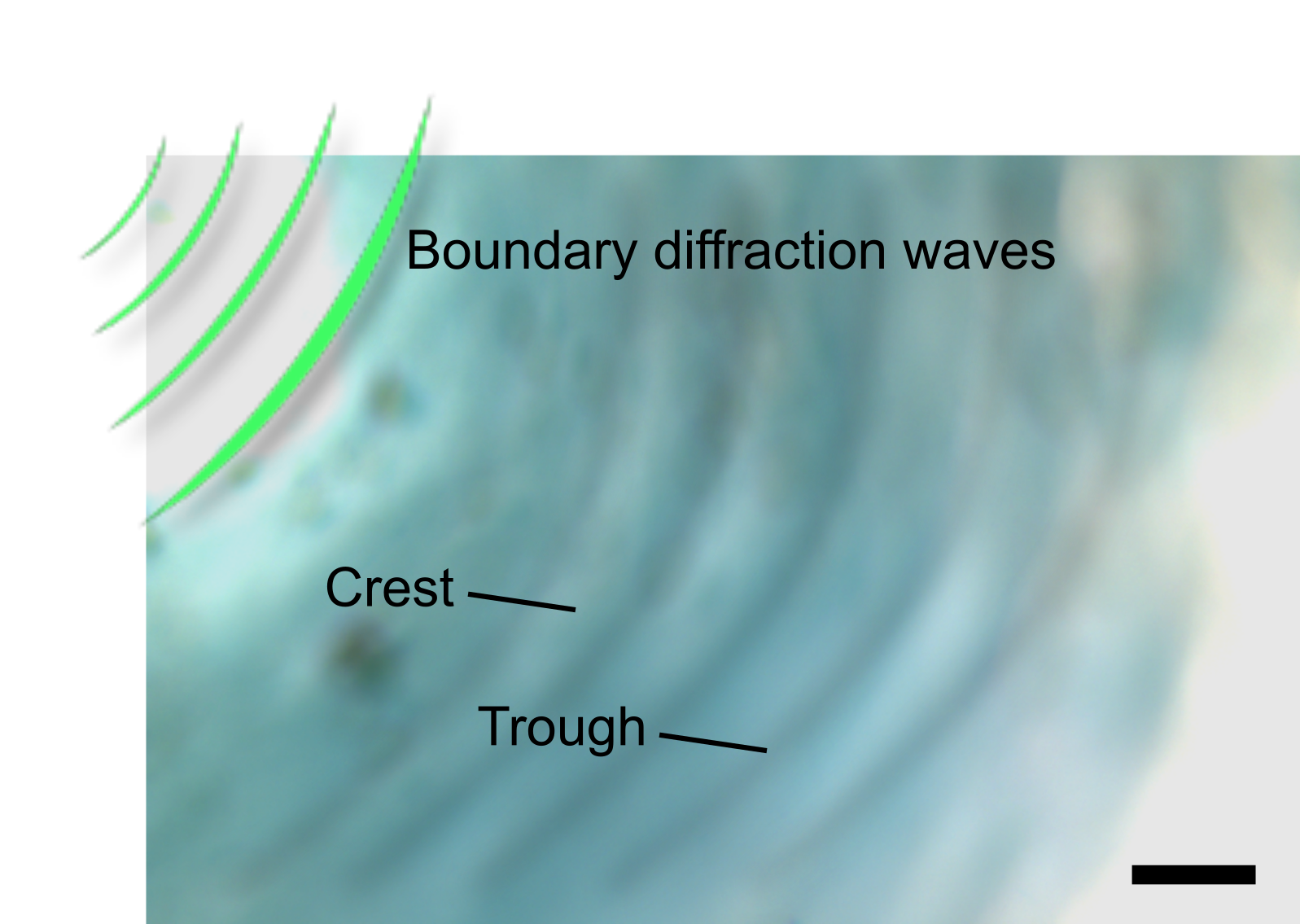

Supplement: Supplementary 1 — Supplementary Methods Figs. S1 to S8 [file research.0263.f1.zip › figs6.png]

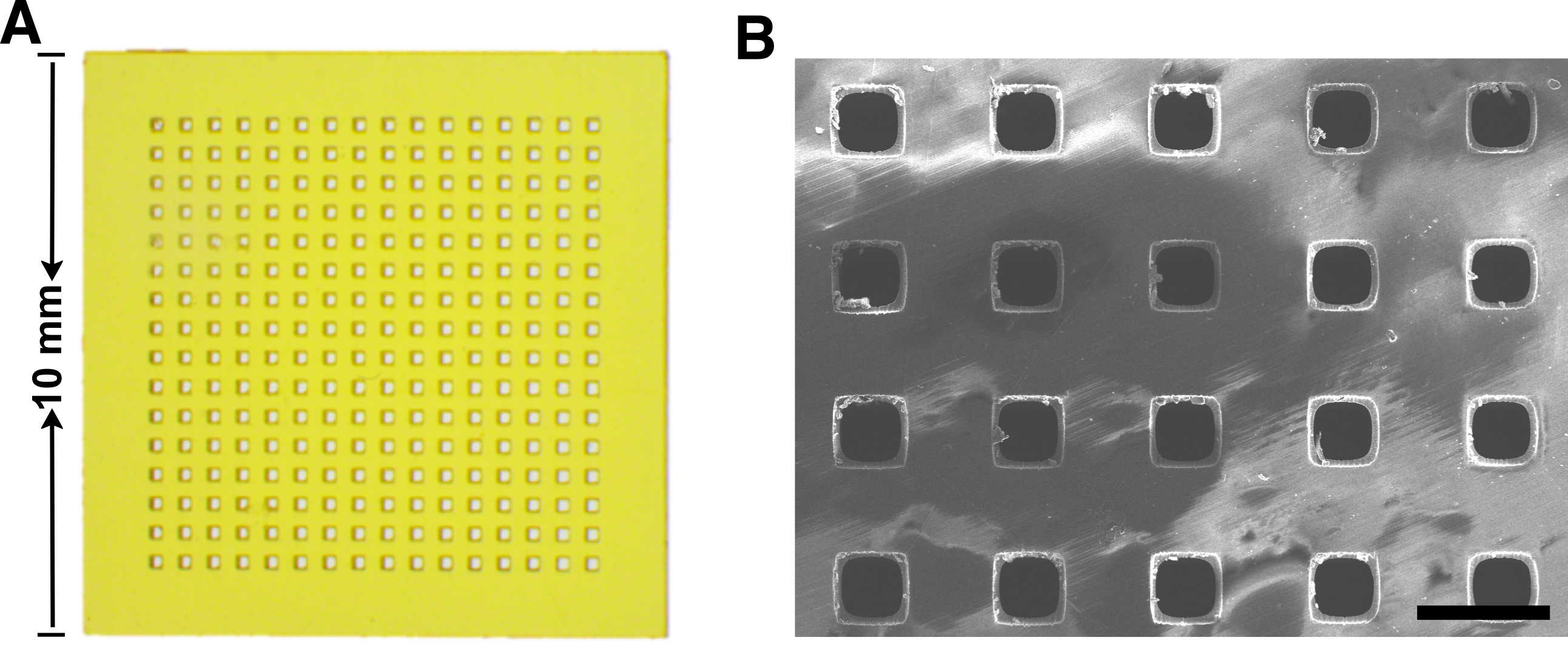

Supplement: Supplementary 1 — Supplementary Methods Figs. S1 to S8 [file research.0263.f1.zip › figs7.png]

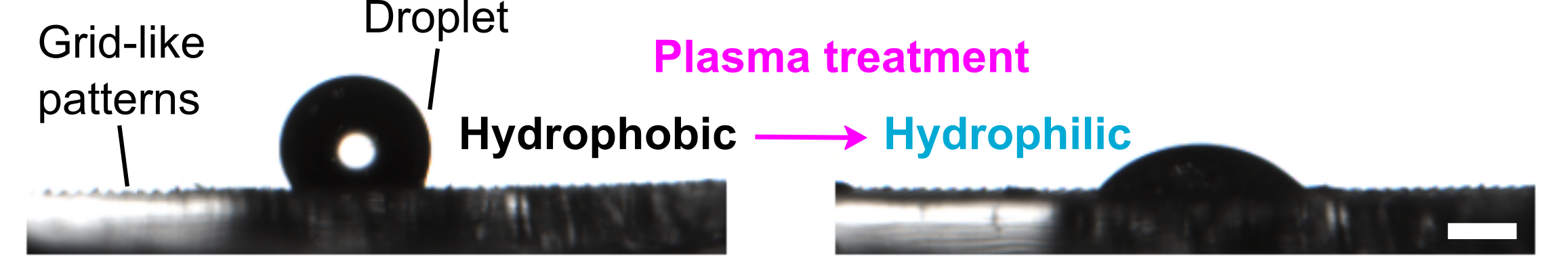

Supplement: Supplementary 1 — Supplementary Methods Figs. S1 to S8 [file research.0263.f1.zip › figs8.png]
